# Conserved and divergent DNA methylation features of extraembryonic tissues between human, mouse and chicken

**DOI:** 10.1186/s12864-026-13123-w

**Published:** 2026-07-06

**Authors:** Georgia Lea, Yuxin Gu, Fengzhu Xiong, Courtney W. Hanna

**Affiliations:** 1 Department of Physiology, Development and Neuroscience, University of Cambridge, Cambridge, UK; 2 Loke Centre for Trophoblast Research, University of Cambridge, Cambridge, UK; 3 Gurdon Institute, University of Cambridge, Cambridge, UK; 4 Institute of Genetics, Zhejiang University School of Medicine, Hangzhou, China

**Keywords:** DNA methylation, Trophoblast, Chorion, Cross-species, Amniotes, Extraembryonic, Methylome

## Abstract

**Background:**

The first cell fate decisions in embryogenesis give rise to extraembryonic tissues, such as the yolk sac and placenta, which are essential for embryo survival. In mammals, extraembryonic cell types arising from these first lineage decisions show distinct DNA methylation patterning, including large partially methylated domains, gene body methylation and accumulation across developmental CpG island (CGI) promoters. Analysis of extraembryonic membranes beyond mammalian species has been limited. Using bisulphite-seq and RNA-seq, we comparatively define these epigenetic characteristics of extraembryonic tissues in both mouse and human. We then tested whether these features are conserved in the chicken extraembryonic membranes (EEMs) including the yolk sac, chorion and chorioallantoic membrane (CAM).

**Results:**

Transcriptomic analysis revealed that embryonic day (E)4 yolk sac/chorion and E14 CAM express similar marker genes to the mouse chorionic trophoblast, supporting that the chicken chorionic ectoderm shares a ‘trophoblast-like’ cell identity. We find that partially methylated domains and transcription-linked gene body methylation patterning are highly conserved between mouse and human proliferative multipotent trophoblast cells but were not evident in the chicken EEMs. However, we find that methylation of CGIs associated with developmental genes was a conserved feature of mouse, human and chicken EEMs.

**Conclusions:**

These findings support that features of the extraembryonic DNA methylation landscape have distinct underlying mechanisms. We find that only one feature is conserved across extant amniotes, suggesting distinct evolutionary pressures are associated with different features of EEM epigenetic landscape.

**Supplementary Information:**

The online version contains supplementary material available at https://doi.org/10.1186/s12864-026-13123-w.

## Introduction

Early embryogenesis is characterised by the specification and development of extraembryonic tissues, such as the yolk sac, amnion and placenta, that support the development of the adjoining embryo. Without these tissues, reproduction fails. However, despite their importance, they have been systematically understudied [1]. Across evolutionary timescales, the extraembryonic membranes of amniote species (reptiles, birds and mammals) have undergone dramatic expansion and regression, their form and function changing drastically to adapt to developmental conditions; most notably with the advent of viviparity to mediate resource exchange between the mother and foetus. In eutherian (placental) mammals, the chorioallantois has expanded to permit viviparity, forming the chorioallantoic placenta [2], while the yolk sac has variably regressed compared with reptiles and birds [3]. Even among mammals, which rely on the placenta for successful reproduction, placental structure and invasiveness varies widely [4]. Beyond morphological comparisons, the molecular mechanisms underlying early cell fate decisions and, importantly, those that underpin the adaptive structure and function of extraembryonic tissues in amniotes remain poorly understood.

One peculiarity of extraembryonic tissues in mammals, is their unique epigenetic patterning, often termed the ‘placental methylome’ when assessed in trophoblast [5, 6]. The main epigenetic feature characterised in this context is DNA methylation, which is the addition of a methyl group onto a cytosine nucleotide, typically within a CpG context. DNA methylation functions to repress gene expression by sterically interfering with DNA-binding proteins or through the recruitment repressive complexes that lead to chromatin compaction [7]. The genome-wide pattern of DNA methylation is broadly similar across embryonic cell types, with high levels of DNA methylation across gene bodies and intergenic regions and low methylation at regulatory elements, such as promoters. The mutagenic capacity of DNA methylation has resulted in a loss of genomic cytosines in vertebrates, leading to unmethylated regions, including 60–70% of promoters, being protected from mutation and consequently resulting in a higher density of CpGs, termed ‘CpG islands’ (CGIs) [8, 9]. In mammals, two notable exceptions to the ‘typical’ DNA methylation landscape are extraembryonic tissues and cancers [10–15].

In eutherian mammals, a hallmark of early development is widespread epigenetic reprogramming, in which DNA methylation patterns are erased and reset. Importantly, re-establishment of genome-wide DNA methylation occurs after trophoblast and hypoblast have been specified; hence, it is anticipated that both trophoblast and hypoblast could have features of this atypical methylome. These features include broad genomic regions with intermediate methylation, termed partially methylated domains (PMDs), and aberrant methylation of polycomb-marked developmental CGIs; although the latter has only been evaluated in mouse trophoblast and hypoblast [5, 12], 16–18]. In addition to PMDs and CGI methylation, mammalian placentas/trophoblasts have been shown to have hypermethylated gene bodies, whose methylation levels correlate with gene expression [5, 6] and widespread placental-specific imprinted regions [19]. Henceforth, we will refer to these features collectively as the ‘extraembryonic methylome.’

The patterning of the extraembryonic methylome in mouse is reliant on the family of *de novo* DNA methyltransferase (DNMT) enzymes [18, 20] and occurs as a consequence of genome-wide epigenetic reprogramming, upon gastrulation [21]. Unexpectedly, a recent study reported that despite the presence of PMDs in marsupial placenta, there is an apparent lack of epigenetic reprogramming during opossum preimplantation development [6, 22]. This suggests that epigenetic reprogramming is not necessarily required for establishment of features of the extraembryonic methylome. Of vertebrates, epigenetic reprogramming during early embryogenesis has only been described in mammals, with studies in zebrafish and xenopus demonstrating that it is not required for development in these species [23]. This raises the question of whether features of the extraembryonic methylome are a mammalian innovation, or are conserved more broadly across amniotes. Indeed, the function of the extraembryonic methylome remains unknown but is hypothesized to permit plasticity in gene regulation and/or co-option of repetitive elements into the gene regulatory landscape [10, 24–26]. Both traits could be necessary and sufficient for the rapid evolution of extraembryonic tissues in therians. To date, the epigenetic patterning of extraembryonic membranes beyond mammals, has not been characterised.

During amniote development there are successive early cell fate decisions that give rise to extraembryonic membranes (EEMs), but as the terminology can vary, we will define the analogous extraembryonic lineages/structures in mammals and oviparous amniotes here for clarity. The first two cell fate decisions during amniote embryo development set aside the outermost chorionic ectoderm-fated cells and the hypoblast (primitive endoderm (PrE) in mice) from the pluripotent epiblast (Epi), which derives the embryo proper. In eutherian mammals, the first lineage is called the trophectoderm, giving rise to chorionic trophoblast lineages that contribute to the chorioallantoic placenta [27]. In oviparous amniotes, this first cell fate, referred to as the ‘area opaca’ in avians and reptilians, gives rise to the chorionic ectoderm of the eventual chorioallantoic membrane (CAM) [28–30]. Across amniotes, the chorioallantois is formed by the fusion of the chorion with the extraembryonic mesoderm-comprised tissue, the allantois, the origin of which remains contentious in some mammals [31]. Some core functions of the chorioallantois have been conserved across amniotes and include foetal blood oxygenation and calcium acquisition [32]. The yolk sac endoderm arises from the second cell fate decision and has remained the site of primitive haematopoiesis and (early) nutrient acquisition either from maternally-provided yolk (oviparous amniotes) or histotrophe (therians), across evolution [33]. Marsupials (metatherians) are an exception, as they do not form a chorioallantois, rather a defining feature of this group is the formation of the choriovitelline membrane, or yolk sac placenta, a membrane comprising chorionic ectoderm of trophoblast origin, extraembryonic mesoderm and hypoblast-derived yolk sac endoderm. Nutrient transport from uterine secretions and/or maternal circulation occurs across the choriovitelline membrane in marsupials [34].

In this study, we aimed to comprehensively define features of the mammalian ‘extraembryonic methylome’ within a single study using data from mouse and human extraembryonic cell types, with a primary focus on proliferative, multipotent trophoblast cells. Analysis of DNA methylation features using whole-genome bisulphite-sequencing showed that PMDs and transcription-linked gene body methylation patterning were highly conserved and well-defined in mouse and human trophoblast cells, as well as DNA methylation at developmental CGIs. Using RNA-seq of chicken EEMs, we demonstrated that E4 yolk sac/chorion and E14 chorioallantoic membrane (CAM) express similar marker genes to the mouse chorionic trophoblast, supporting an analogous ‘trophoblast-like’ cell identity of chorionic ectoderm. We then assessed features of the mammalian extraembryonic methylome in chicken EEMs. While PMDs and transcription-associated gene body methylation were not conserved features in chicken, methylation of CGIs associated with developmental genes was a conserved feature of human, mouse and chicken chorionic trophoblast/ectoderm cells.

## Results

### Defining the extraembryonic methylome in mouse and human proliferative trophoblast

**Fig. 1:**
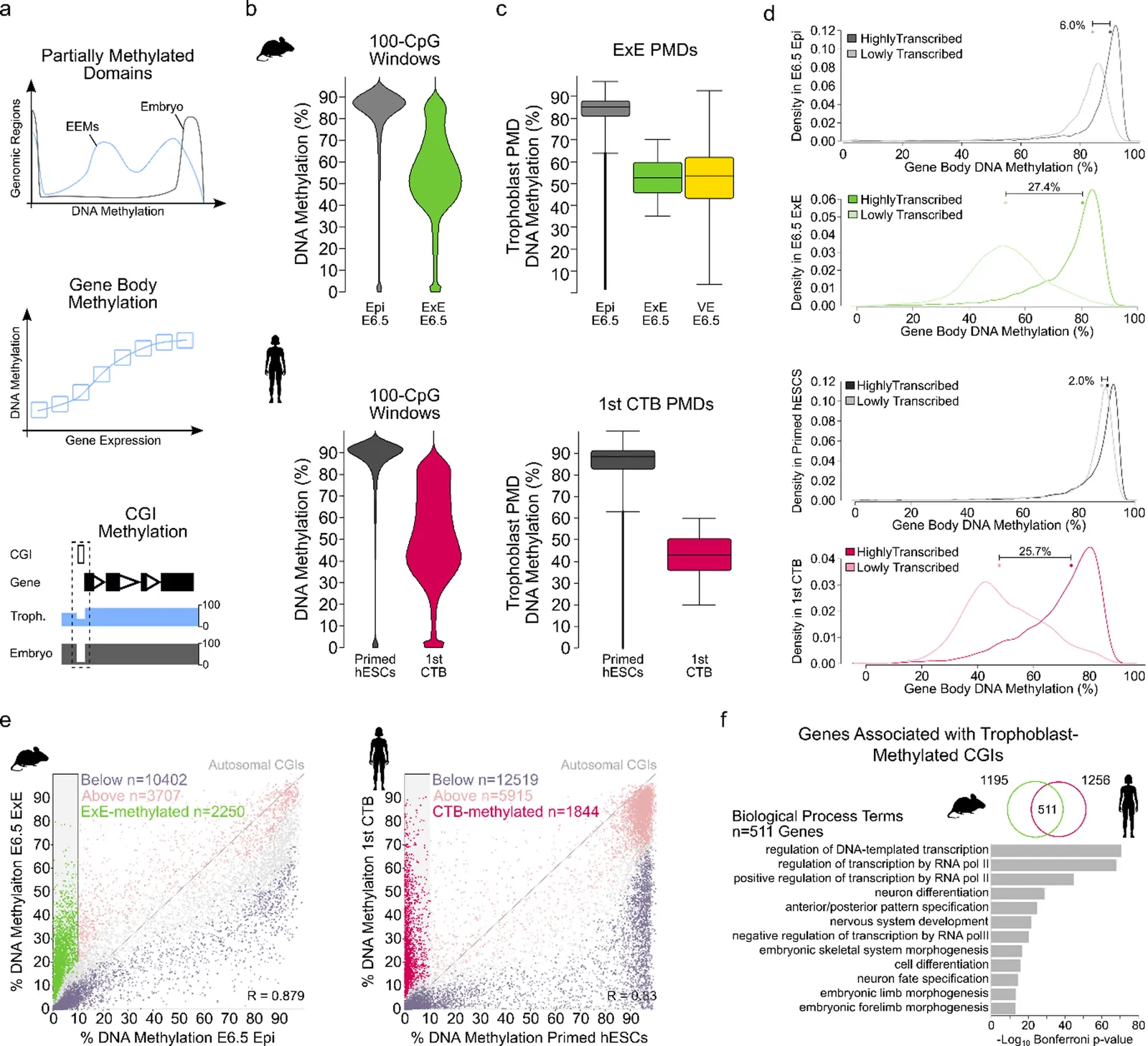
Quantitatively defining features of the mammalian extraembryonic methylome.** a** Schematic depicting the three hallmark features of the mammalian extraembryonic methylome assessed in this study: (1) the distinct genome-wide distribution of DNA methylation in extraembryonic cells compared to somatic/epiblast-derived cells including the characteristic partially-methylated domains (PMDs) (top); (2) the correlation of gene body methylation with gene expression level (middle); and (3) the hypermethylation of a subset of CGIs at the promoters of developmental genes (bottom). **b** Bean plots showing genomic distribution of DNA methylation for 100-CpG windows in mouse (top) and human (bottom) representative pluripotent embryonic cells (E6.5 Epi and primed human embryonic stem cells (hESCs), respectively) compared to isolated proliferative, multipotent trophoblast cells (E6.5 ExE and 1st cytotrophoblast (CTB), respectively). **c** Box and whisker plots showing DNA methylation of trophoblast-defined PMDs in each tissue type in mouse (top) and human (bottom). **d** Density plots showing DNA methylation of genes in mouse (top) E6.5 Epi and ExE and human (bottom) primed hESCs and 1st CTB. Genes were divided into two groups, highly or lowly transcribed, defined as RPKM > 1 and RPKM < 1 in each tissue, respectively. The difference in median % methylation (filled circle) between each group is displayed. **e** Scatter plots showing differentially methylated CGIs in mouse E6.5 ExE compared to Epi (left) and human 1st CTB compared to primed hESCs (right). ExE-methylated CGIs (boxed, green) were defined as above the distribution, hypermethylated in ExE and < 10% methylation in E6.5 Epi. Human 1st CTB-methylated CGIs (boxed, magenta) were defined as above the distribution, hypermethylated in CTB, with < 10% methylation in primed hESCs. **f** Venn diagram showing common genes associated with trophoblast-methylated CGIs in both mouse and human (top). The top 12 terms from Gene ontology (GO) analysis of shared trophoblast-methylated CGI-associated genes (*n* = 511) is shown (bottom)

Although aspects of the extraembryonic methylome have been described in various species [5, 6, 12, 18, 35, 36], its distinctive features have not been comprehensively defined in both mouse and human within a single study (Fig. 1a). Thus, to interrogate whether mammalian extraembryonic DNA methylation patterning is present in *G. gallus*, we first aimed to clearly define these features in extraembryonic cell types from mouse and human. Notably, due to a lack of allelic resolution and a reported absence in chicken, we did not assess epigenetic patterns linked to dosage compensation, such as sex chromosome inactivation or genomic imprinting [37, 38].

Global patterning of DNA methylation was assessed for 100-CpG windows across autosomes in the *M. musculus* (GRCm38) and *H. sapiens* (GRCh38) genomes, with a minimum coverage of 10 CpGs. For these analyses we compared published data for proliferative trophoblast cells from mouse (E6.5 extraembryonic ectoderm (ExE), *n* = 2) [12] and human (1st trimester cytotrophoblast (1st CTB), *n* = 2) [39], as well as mouse E6.5 visceral endoderm (VE, *n* = 2) which arises from PrE [18]. These extraembryonic cell types were compared to pluripotent embryonic cells in each species; in mouse, E6.5 Epi (*n* = 2) [12] and, in human, primed embryonic stem cells (primed hESCs, *n* = 3) [40] (Tables S1, S2). Mammalian extraembryonic cells were globally hypomethylated, with mouse E6.5 ExE trophoblast showing an average methylation of 54.3% and E6.5 VE an average of 53.5% compared to 77.5% in E6.5 Epi (*p* < 0.0001 and *p* < 0.0001 (two-tailed t-test), respectively) (Fig. 1b). Similarly, human 1st CTB showed an average methylation of 51.5% compared to 81.0% in primed hESCs (*p* < 0.0001, two-tailed t-test) (Fig. 1b).

We first assessed the global distribution of DNA methylation in mouse and human trophoblast. Embryonic tissues in both mouse and human showed the classical bimodal distribution, with regions of the genome being either highly methylated or unmethylated (Fig. 1b). This contrasted with the distinctive trimodal distribution of proliferative trophoblast cells, in which the majority of the genome is partially methylated (Fig. 1b). PMDs were then defined separately in each species based on the data distribution; in mouse, PMDs were defined as methylation > 35% and < 70% and in human, PMDs were defined as previously, > 20% and < 60% [41] (Fig. 1c). In mouse E6.5 ExE, PMDs corresponded to 67.6% of 100-CpG windows, while in human 1st CTB, PMDs accounted for 56.0%. Due to the relative low read depth of the E6.5 VE data, we could not define PMDs within this tissue, but upon assessing the methylation levels of informative 100-CpG windows within ExE PMDs, we found a similar mean methylation level (VE = 52.5%, ExE = 52.6%) supporting that these lineages have broadly similar genomic distributions of DNA methylation (Fig. 1c). In addition to PMDs in both mouse and human trophoblast, we observed a significant, positive correlation between gene expression and gene body DNA methylation (Fig. S1a-b) (mouse *R* = 0.72, *p* < 0.0001; human *R* = 0.59, *p* < 0.0001, Pearson’s correlation). To define the pattern of gene body methylation within extraembryonic lineages for later comparative analyses, we plotted the distribution and median of gene body methylation at highly expressed (RPKM > 1) and lowly expressed (RPKM < 1) genes, defined within each cell type. Mouse E6.5 ExE and human 1st CTB showed a > 25% difference in median methylation between high and lowly expressed genes (27.4% and 25.7%, respectively), whereas E6.5 Epi and primed hESCs showed very subtle differences (6.0% and 2.0%, respectively) (Fig. 1d).

The final feature of the mammalian extraembryonic methylome is DNA methylation at a subset of developmental CGIs, which has been previously characterised in mouse [12, 18]. DNA methylation was quantitated over CGIs [42] with a minimum of 10 informative CpGs. We compared mouse E6.5 ExE to Epi, to obtain a list of 2250 hypermethylated CGIs in E6.5 ExE, termed “ExE-methylated CGIs” (Fig. 1e). We then performed this analysis for human CGIs (Ensembl annotation), comparing 1st CTB to primed hESCs. We identified 1844 human CTB-methylated CGIs in 1st CTB (Fig. 1e). To compare the conservation of these trophoblast-methylated CGIs, we identified genes within 2 kb of these CGIs (*n* = 1195 in mouse, *n* = 1256 in human). A set of 511 genes associated with trophoblast-methylated CGIs were shared between mouse and human. Gene ontology (GO) analysis of these genes identified terms linked to development, embryo patterning and transcriptional regulation (Fig. 1f, Table S3), consistent with these regions being Polycomb targets, as has been previously reported in mouse [12]. Together, these analyses demonstrate that all features of the extraembryonic methylome are quantifiable and highly similar between mouse and human trophoblast cells.

### Conservation of lineage-specific markers between analogous extraembryonic membranes in mouse and chicken embryos

**Fig. 2:**
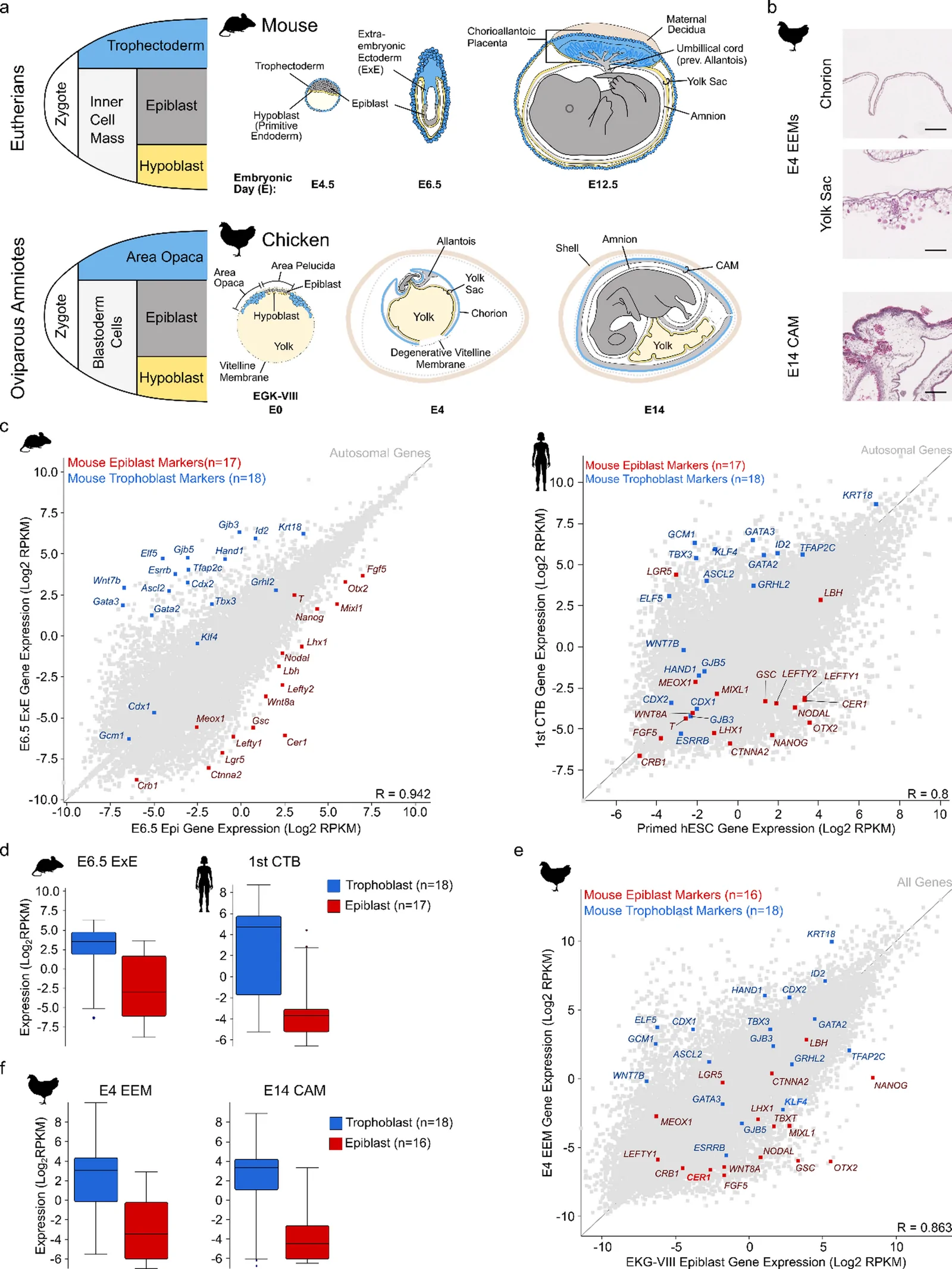
Chicken chorion-containing E4 EEM and E14 CAM express mammalian trophoblast marker genes.** a** Schematic illustrating the common origin of extraembryonic tissues in mouse (top) and chicken (bottom) in the first three cell fate decisions. The development of each species is depicted (not to scale) from early development (left) to late development (right) with labelled embryonic day (E) of development. For E0 chicken embryo, EGK-VIII refers to Eyal-Giladi and Kochav stage 8 of intrauterine development. Relevant structures are labelled and tissues are colour-coded based on origin of cell type in the first three cell fates, namely extraembryonic ectoderm in blue, hypoblast-derived endoderm in yellow and epiblast-derived layers and/or structures in grey. Chicken chorion consists of chorionic ectoderm (blue) and extraembryonic mesoderm (grey). Both mouse and chicken yolk sac consist of hypoblast-derived endoderm (yellow) and extraembryonic mesoderm (grey). The chicken chorioallantoic membrane (CAM) is formed by the fusion of the allantois with the chorion. **b** H & E staining of chicken extraembryonic membranes collected at each time point; E4 chorion and yolk sac (top) and E14 CAM (bottom). Scale bar is 150 μm. **c** Scatterplot comparing autosomal gene expression (Log_2_RPKM) in mouse E6.5 Epi and E6.5 ExE (left) and human primed hESCs and 1st CTB (right). Expression of mouse Trophoblast (*n* = 18) and Epiblast (*n* = 17) marker genes are highlighted in blue and red, respectively. **d** Box and whisker plots showing high levels of gene expression (Log_2_RPKM) of mouse trophoblast (blue) and low expression of mouse epiblast (red) marker genes in mouse E6.5 ExE and human 1st CTB. **e** Scatterplot comparing gene expression (Log_2_RPKM) of chicken EGK-VIII epiblast-equivalent cells from the area pellucida and E4 EEM, highlighting expression of murine trophoblast genes (blue) and mouse epiblast markers (red). *KLF4* and *CER1* are highlighted in bold as these reside on chicken sex chromosome, Z. (Note, *LEFTY2* is not annotated in the chicken genome.) **f** Box and whisker plots showing high levels of gene expression (Log_2_RPKM) of mouse trophoblast (blue) and low expression of mouse epiblast (red) marker genes in chicken E4 EEMs and E14 CAM

Chicken is a widely used model organism for developmental studies and hence its EEMs have been well-characterised. To initially explore the similarities in cell identities between extraembryonic tissues arising from the first lineage decision in the chicken to those in mouse and human (Fig. 2a), we collected EEMs from E4 chicken embryos, including the chorion and yolk sac (referred to collectively as EEMs) (*n* = 3), and the fused chorioallantoic membrane at E14 (*n* = 2) and performed stranded RNA-sequencing (Fig. 2a-b). These data were compared to published RNA-seq data from stage EGK-VIII, multipotent chick area pellucida (*n* = 2) [43], representing an epiblast-equivalent cell population (Fig. 2a). These datasets were then contrasted with mouse E6.5 ExE trophoblast (*n* = 2) and Epi (*n* = 2) [44], and sorted human 1st CTBs (*n* = 3) [39] and primed hESCs (*n* = 3) [45] (Table S1).

We first confirmed that reported markers of murine multipotent and chorionic trophoblast, such as *Wnt7b*, *Grhl2*, *Eomes* and *Esrrb* [46], were indeed lineage-specific in mouse E6.5 embryos, being highly expressed in ExE compared to Epi at E6.5 (Fig. 2c-d). These marker genes showed consistent lineage-specific expression in human, comparing 1st CTB to primed hESCs (Fig. 2c-d). Remarkably, despite their highly diverged morphology, function and over 300 million years of evolution, several key mouse trophoblast marker genes were highly expressed in chicken E4 EEMs compared to EGK-VIII epiblast (Fig. 2e-f). Consistently, these genes maintained their expression in the chicken E14 CAM (Fig. 2f). Together, these data illustrate that despite the mixed cell populations present in chicken EEMs, they exhibit a robust trophoblast-like transcriptional signature consistent with the shared origin of the chorionic ectoderm from the first cell fate decision.

### Conserved methylation of CGIs in chicken extraembryonic tissues

To define features of the extraembryonic methylome in chicken, we performed whole-genome post-bisulphite adaptor tagging (PBAT-) sequencing on biological replicates of chicken E4 EEMs (*n* = 3) and E14 CAM (*n* = 4). Due to the challenges of collecting epiblast-equivalent (area pellucida) cells in chicken, we collected E8 embryonic brain (*n* = 3) for PBAT-sequencing, as a proxy for epiblast-derived tissue. To validate this approach, we compared the methylation signature between mouse E6.5 Epi and postnatal day 1 (P1) brain [47]. Consistently, P1 brain showed a high global average methylation (72.0%) and low mean DNA methylation levels at ExE-methylated CGIs (3.3% in P1 brain versus 3.4% in E6.5 Epi) in contrast to E6.5 ExE (22.9%) (Fig. 1e, Fig. S2a).

**Fig. 3:**
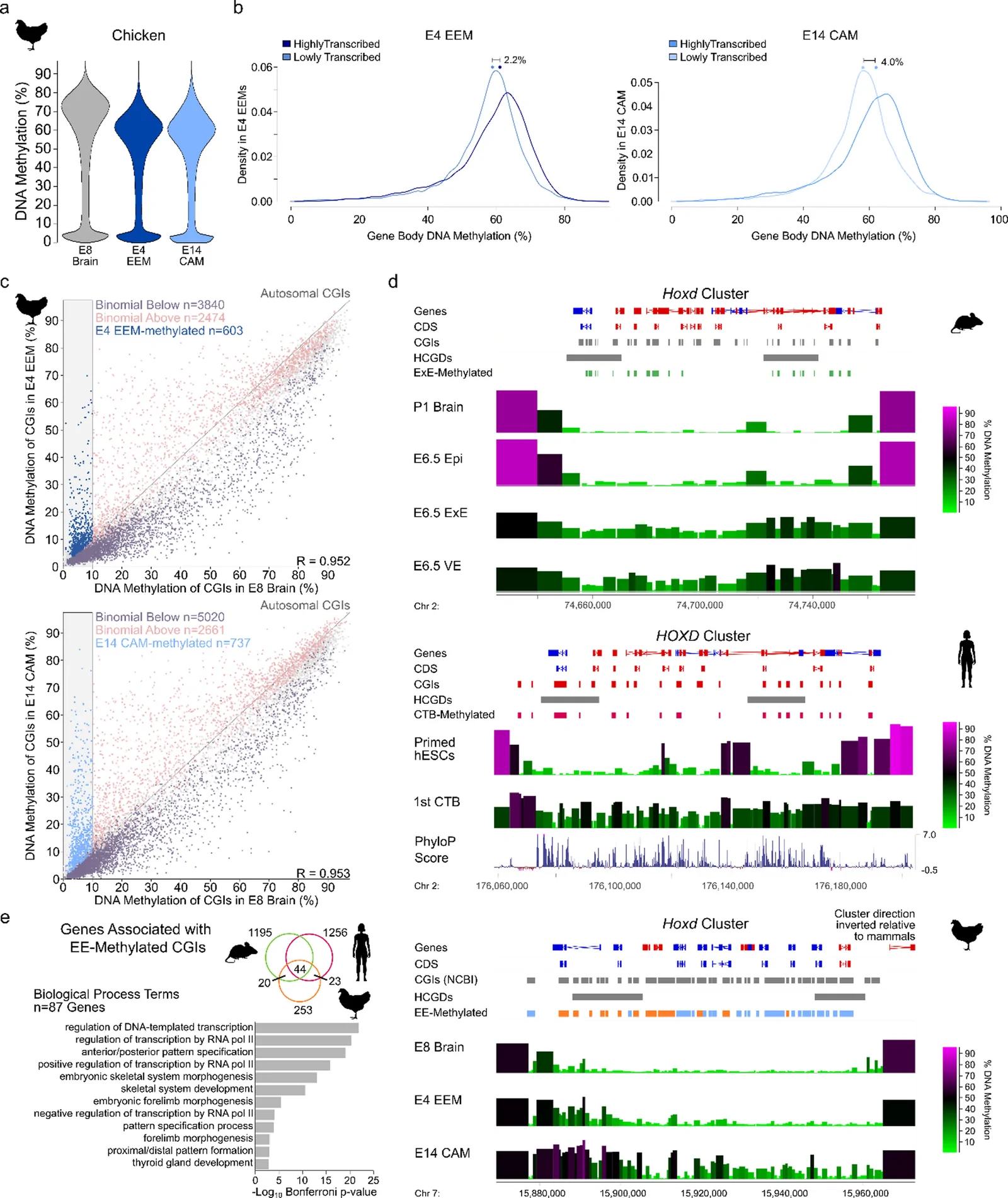
Chicken EEMs show conservation of hypermethylation at CGIs associated with developmental genes. **a** Bean plots showing similar bimodal distribution of DNA methylation in chicken E8 brain, E4 EEMs and E14 CAM. **b** Density plots showing gene body DNA methylation of highly transcribed (RPKM > 1) and lowly transcribed (RPKM < 1) genes in E4 EEM (left) and E14 CAM (right). The median is displayed as a coloured point, and the difference in medians between the two groups is labelled. **c** Scatter plots comparing DNA methylation of autosomal CGIs in E8 brain compared to E4 EEMs (top) and E14 CAM (bottom) to define EE-methylated CGIs in chicken (*n* = 603 and *n* = 737, respectively), using the same criteria as mouse and human (Fig. 1). **d** Screenshots showing DNA methylation of 100-CpG windows over the *HOXD* gene cluster in each species, mouse (top), human (middle) and chicken (bottom). The trophoblast-methylated CGIs within the cluster are shown for mouse (green) and human (magenta) and EE-methylated CGIs in chicken are highlighted with E14 CAM-specific CGIs in blue and those methylated in both E4 and E14, in orange. PhyloP score across the region is shown for the human *HOXD* cluster and the location of two orthologous highly conserved genomic domains (HCGDs) [50] are shown for each species. **f** Venn diagram showing overlap of genes associated with EE-methylated CGIs across mouse, human and chicken tissues (top). Bar chart displaying top 12 terms resulting from GO analysis on the list of EE-methylated CGI-associated genes identified in chicken and mouse and/or human (*n* = 87) (bottom)

Chicken E4 EEMs and E14 CAM had globally similar mean DNA methylation levels at 45.8% and 46.5%, respectively, which was 5.5–6.2% lower than the corresponding embryonic tissue, E8 brain, at 52.0% (*p* < 0.0001, *p* < 0.0001 (two-tailed t-test), respectively). These data corroborate others’ findings that global DNA methylation levels in avian tissues are lower compared to eutherian mammals [48, 49]. In contrast to mammalian extraembryonic cell types, which were globally hypomethylated by > 20% compared to embryonic cells, the difference in chicken was relatively subtle. Furthermore, all chicken tissues had a bimodal distribution of DNA methylation with no evidence of a defined subset of intermediately methylated genomic regions (Fig. 3a, Fig. S2b). Next, we assessed the link between gene transcription and genic DNA methylation in chicken EEMs. In chicken E4 EEM and E14 CAM, the difference in gene body methylation between high and lowly expressed genes was extremely subtle (< 5%) (Fig. 3b), similar to that observed in mammalian embryonic lineages (Fig. 1d). Correlations between gene expression and gene body methylation similarly showed subtle links (Fig. S2c) (E4 *R* = 0.04, *p* < 0.0001, E14 *R* = 0.11, *p* < 0.0001, Pearson’s correlation). Taken together, these data support that gene body DNA methylation correlating with expression and PMDs are not conserved in chicken EEMs and thus, likely to be mammalian-specific features of the extraembryonic methylome.

To investigate whether there were EE-methylated CGIs in the chicken, we quantified DNA methylation over CGIs (NCBI annotation) with at least 10 informative CpGs in E4 EEMs and E14 CAM, comparing these to E8 brain. Using the same approach as mouse and human (detailed in Materials and Methods), we identified E4 EEM-methylated CGIs (*n* = 603) and E14 CAM-methylated CGIs (*n* = 737) (Fig. 3c). A set of 368 CGIs, which were found to be hypermethylated in extraembryonic tissues at both time points, was used for further analysis and termed “common EE-methylated CGIs” (Fig. S2d). Notably, these common EE-methylated CGIs gained methylation between E4 (mean = 15.5%) and E14 (mean = 22.0%) (Fig. S2e). It has been described previously, that mammalian extraembryonic-methylated CGIs reside in regions of lower-than-average DNA methylation in embryonic tissues, termed DNA methylation valleys [18]. Interestingly, this aspect of EE-methylated CGIs was also conserved across species (Fig. 3d). Genome browser screenshots showing DNA methylation within 100-CpG windows across the *HOXD* gene cluster in each species exemplifies the conservation of these features in amniote EEMs (Fig. 3d). Notably these regions do not just show conservation at the epigenetic level, the high PhyloP score across the region reflects its sequence conservation, and many of the EE-methylated CGIs within this cluster overlap annotated Polycomb-targeted, highly-conserved genomic domains (HCGDs) [50].

Finally, we identified genes within 2 kb of the 368 common EE-methylated CGIs in chicken, which were then filtered for those with orthologues across all three genomes (*n* = 253). These 253 EE-methylated CGI-associated genes were then compared across species (Fig. 3e). Strikingly, 34.4% of genes associated with EE-methylated CGIs in chicken were similarly associated with an EE-methylated CGI in either mouse or human, or both (*n* = 87, Fig. 3e). Gene Ontology analysis of these conserved EE-methylated CGI-associated genes showed a strong enrichment for terms related to development, embryo patterning, and transcriptional regulation (Fig. 3e, Table S4). It has been previously reported that methylated CGIs within mouse ExE are associated with gene repression and closed chromatin [12]. Consistently, we observed that genes associated with EE-methylated CGIs are predominantly lowly expressed in mouse and human, with a smaller majority showing low expression in chicken (Fig. S2f). Together, these findings support that there is a conserved signature of EE-methylated CGIs associated with developmental genes in mouse, chicken and human extra-embryonic tissues.

## Discussion

In this study, we define features of the mammalian extraembryonic DNA methylation landscape in mouse and human proliferative trophoblast cells, and evaluated whether these features were conserved in chicken chorion/yolk sac and CAM. The presence of widespread PMDs and transcription-coupled gene body methylation were absent in chicken. Yet, remarkably, methylation of a subset of CGIs associated with developmental genes was conserved across human, mouse and chicken. While the number of methylated CGIs was lower in chicken and many of the local genes were distinct, these broadly encompass a group of developmental transcriptional regulators, a feature that is conserved across mouse, human and chicken. The conservation of only a single feature of the extraembryonic DNA methylation landscapes reveals that this feature likely has distinct mechanistic underpinnings. Furthermore, this finding supports that CGI methylation may have a functional role in genomic regulation being either an ancestral trait or having convergently evolved in extant amniotes.

Using comparative embryology, the mammalian placenta and chicken CAM are reported to be highly similar in both structural composition and cellular origin [51, 52], yet similarities in cellular identities have not been molecularly characterised. Using transcriptomic analysis, we have shown that there is conserved expression of mouse multipotent and chorionic trophoblast marker genes in chicken EEMs, likely reflecting the analogous cell origin of the chicken chorionic ectoderm to mammalian trophoblast in the first lineage decision during embryogenesis. A limitation of this study is the use of whole tissue dissected from chicken embryos, compared to isolated mammalian trophoblast and visceral endoderm populations. Nonetheless, a trophoblast transcriptional signature was evident despite the presence of contaminating populations such as extraembryonic mesoderm and embryonic blood, in day 14 CAM.

Importantly, the finding that PMDs are not conserved in *G*. *gallus* EEMs could support that extraembryonic PMDs are indeed a mammalian innovation, along with other mammalian-specific epigenetic processes such as genomic imprinting and X chromosome inactivation [53, 54]. Notably, the low global average [48, 49] and lack of PMDs/transcription-associated gene body DNA methylation in chicken may suggest altered *de novo* DNMT-histone interactions in birds. This could be because of or in complement to the lack of global reprogramming, which may warrant future investigation. Alternatively, it is possible that the evolutionary origin of extraembryonic PMDs resides earlier in amniote evolution and PMDs have merely been lost in the Archelosauria branch of reptiles, or other sub-clade to which birds belong. It is likely relevant that viviparity has not yet been described in any species of Archelosauria, while this mode of reproduction is pervasive among the sister order of reptiles, Squamata [55–58]. To test whether the plasticity of reproductive mode is reflected in the epigenetic profile of the tissues at the foeto-maternal interface, it would be valuable to profile DNA methylation of closely related viviparous and oviparous species of snakes, for example.

Intriguingly, global DNA hypomethylation in the form of PMDs is also reportedly a common feature of many cancers. It has been hypothesized that PMDs may confer epigenetic plasticity and the widespread co-option of repetitive elements into the gene regulatory landscape [10], 24–26]. The capacity for dynamic genomic regulation may therefore underpin the responsiveness of both the placenta and cancers to varying physiological conditions of the surrounding tissues, such as changes in oxygenation and nutrient supply [59]. However, an anticipated consequence of this prolonged widespread hypomethylation is genomic instability, which is not only a hallmark and driver of cancer [60], but a feature of placental trophoblast cells in vivo and in vitro [61, 62].

The vast majority of CGIs are unmethylated in almost all cellular contexts due to the exclusion of DNMTs by the presence of histone 3 lysine 4 trimethylated (H3K4me3)-marked nucleosomes. In a developmental context, hypermethylation of developmental CGIs has been best characterised in mouse ExE [12] and VE [18]. Given the lack of conservation of PMDs in chicken EEMs, it was perhaps an unexpected finding that hypermethylation of developmental CGIs is conserved. Additionally, we noted that those CGIs identified at both time points in chicken extraembryonic tissues, gained methylation during development. We were unable to comment on the methylation dynamics of these CGIs across gestation in mouse and human. Survey of these CGIs during gestation and the dynamics of expression of the associated genes, warrants future study.

Notably, hypermethylation of developmental CGI promoters is also a pan-cancer epigenetic signature, although the underlying mechanisms and functions remain poorly understood [63]. Genetic manipulations of chromatin-interacting domains of the DNMT enzymes, with a predominant focus on DNMT3A, has shed some light on potential underlying mechanisms. DNMT3A is a highly conserved protein with an ATRX-DNMT3A-DNMT3L (ADD) and Pro-Trp-Trp-Pro (PWWP) domain, which bind unmodified H3K4 and H3K36me2/3, respectively. DNMT3A-mediated *de novo* methylation in post-implantation embryos is directed predominantly through the PWWP domain interactions [64], but interaction of DNMT3A with catalytically inactive cofactor DNMT3L alters this affinity [65]. Further, mutations in the DNMT3A PWWP domain have been described to cause aberrant DNA methylation accumulation over developmental, Polycomb-marked CGIs in embryonic lineages [47, 66]. The establishment of DNA methylation in mouse and human trophoblast has been shown to be at least partially dependent upon DNMT3L [20, 41]. Hence, DNMT3L expression in mammalian extraembryonic tissues may alter the faithful exclusion of DNA methylation from CGIs. However, our finding of hypermethylation of developmental CGIs in chicken, which lacks an orthologous *DNMT3L* gene [67], support this may be a DNMT3L-independent mechanism, or that a homologue, such as catalytically inactive DNMT3B3 [68], may perform a similar function in chicken EEMs. Future studies will help delineate the mechanisms that license DNMTs to target these CGIs in a lineage-specific way and, furthermore, whether this epigenetic state plays a functional role in gene regulation and development.

## Conclusions

With this study, we present a bioinformatic approach to comprehensively define conserved features of the extraembryonic methylome and use this approach to investigate their evolutionary conservation. We have shown that despite millions of years of evolution and vastly divergent structure and functions, the mammalian trophoblast transcriptional signature is expressed in chorionic tissues of chicken, the ectoderm of which is derived from the first cell fate decision in early amniote embryogenesis. We also present the first genome-wide characterisation of DNA methylation in chicken EEMs. Our analysis shows that PMDs and gene body hypermethylation are absent from chicken, supporting that these have coevolved with expanded function of chorionic membranes in placental mammals. Critically, we have identified the conservation of DNA methylation at CGIs associated with developmental transcriptional regulators in extraembryonic tissues of humans, mouse and chicken, which has intriguing mechanistic and functional implications. Given the apparent similarities between extraembryonic tissues and cancer, this approach will be valuable for future comparative and functional studies to gain a better understanding of these atypical epigenomes.

## Materials and methods

### Sample collection

All procedures comply with the UK Animals (Scientific Procedures) Act 1986, which states that procedures performed on embryonic stages (under 2 weeks, or 2/3 of gestation time for chicken) do not require an animal protocol.

Chicken (*Gallus gallus*) eggs were supplied by Medeggs Inc. Eggs were stored in 15 °C fridge, then incubated in a vertical position with the wider end on top at 37.5 °C in ~ 60% humidity incubators (Brisnea) for 4, 8, and 14 days. Day 8 and 14 embryos were decapitated immediately after egg opening and all egg materials were disposed following an institute autoclave protocol of biological materials at Gurdon institute.

For embryonic day 4 (E4) chorion, a window was opened on the wider end and the shell membrane was removed before dissection. At this stage the extraembryonic coelom has not expanded yet and the chorion is closely in touch with the blood vessels (extraembryonic mesoderm lineage) and the yolk sac, with chorion on the surface and the yolk sac inside. This composite tissue was carefully dissected and left in an intact state which mimics the CAM in terms of tissue types. It was then placed in ice-cold PBS to be unfolded and washed to clear the albumen. The dissected area is lateral to the embryo to ensure no embryonic tissues or amnion included. Samples from each egg were cut into 3 roughly equal-sized pieces, each piece was then stored at -80 °C in 0.2 ml PCR tube with minimal PBS.

For E14 CAM, the egg was opened from the long side and the embryo was poured out, leaving the CAM attached to the shell. The CAM was then peeled off from the shell with intact chorioallantois, chorion layers and blood vessels. The tissue was cut into 3 pieces, placed separately in 1.5 ml Eppendorf with minimal PBS, and stored at -80 °C.

For E8 brain, the egg was opened from the long side. The embryo was decapitated and the head was washed in ice-cold Ringer’s solution. Overlaying tissues (chorion, amnion and skin) were first carefully removed from the head, exposing the brain, and 2 sections of the brain were collected, washed in PBS, and stored at -80 °C in 0.2 ml PCR tube with minimal PBS.

### Histological analysis

Samples for histology were collected following the same dissection protocols. They were fixed in 4% PFA at 4 °C overnight, and washed 5 min, 3 times with PBS the next day. Following fixation, samples were dehydrated with ethanol before being incubated overnight in Xylene. Samples were then paraffinized and embedded. Paraffin blocks were then sectioned in 8 μm sections. Selected sections were then deparaffinised before staining with haematoxylin and eosin (H & E), using standard protocols. H & E stained sections were then imaged using a Nanozoomer (Hamamatsu).

### Bisulphite-sequencing library generation

Low input bisulphite-sequencing libraries were generated, as previously described [69], using a post-bisulphite adaptor tagging (PBAT) method. DNA extraction for E14 CAM samples (n = 4) was performed with QIAamp Fast DNA Tissue Kit (Qiagen), per the manufacturers’ protocol. E4 EEM (n = 3) and E8 Brain (n = 3) samples were thawed on ice before lysis in 10mM Tris buffer with 0.5% SDS and proteinase K for 2 hours at 37°C. Bisulphite conversion was performed using the EZ DNA Methylation Direct Kit (Zymo Research) or Imprint DNA methylation kit (Sigma; for E14 CAM 3.1 and 5.3), as per the manufacturers’ protocol. Following elution, first strand synthesis was performed with Klenow Fragment (3’-5’ exo-) enzyme (New England Biolabs) and customised 9 bp random sequence containing biotin-conjugated adaptor (PBAT-9N-F) after which Exonuclease I (New England Biolabs) was added and incubated for 1 h at 37 °C. SPRI bead purification was then performed to remove unincorporated adaptors, with library fragments captured using Dynabeads M-280 Streptavidin (Thermo Fisher Scientific). Second strand synthesis was performed on DNA-bound beads using Klenow Fragment (3’->5’ exo-) enzyme (New England Biolabs) with customised 9 bp random sequence containing biotin-conjugated adaptor (PBAT-9N-R). After washing DNA-bound beads with EB buffer (Qiagen), libraries were amplified with Phusion High-Fidelity DNA polymerase (New England Biolabs) in 50 µL reaction for 10 cycles using PE1.0 and PE2.0 with an indexing barcode for each library. Libraries were then purified using SPRI beads and quantitated using the High DNA Sensitivity Bioanalyzer 2500 (Agilent) and Illumina library quantification kit (KAPA). Samples were multiplexed for sequencing on an Element AVITI sequencer, using 75 bp paired-end mode. Samples and sequencing depths for each library is provided in Table S2.

### RNA-sequencing library generation

RNA-sequencing libraries were generated as previously described [70]. RNA extraction from snap-frozen E14 CAM tissue was performed in 400µL TRIzol (Invitrogen), and phase-separated by adding 80µL of chloroform: isoamyl alcohol (Sigma-Aldrich) following centrifugation for 15 min at 4 °C. The aqueous phase was added to a mixture of 200µL isopropanol with 1µL GlycoBlue and incubated on ice for 10 min. The RNA was then pelleted by centrifugation at 4 °C for 10 min, after which the pellet was washed with 75% ethanol. The pellet was then airdried and resuspended in 5µL RNase-free water. Using the mRNA DIRECT kit (Thermo Fisher Scientific) with adaptations for low input, as previously described, mRNA capture was performed with Oligo (dT)25 Dynabeads. Libraries were then generated using the SMARTer Stranded RNA-seq kit (Takara) from isolated mRNA, as per the manufacturers’ guidelines, using 12 amplification cycles. Libraries were sequenced using 50 bp single-end mode on an Element AVITI sequencer. Samples and sequencing depths for each library is provided in Table S2.

### Publicly available data

Published mouse datasets utilised in this study are summarised in Table S1. Mouse datasets include PBAT-seq data for E6.5 ExE and Epi (GSE84236) [12] and VE (GSE76505) [18] and RNA-seq data for E6.5 ExE and Epi (GSE97778) [44]. Human datasets included in this study are, PBAT-seq and RNA-seq for 1st trimester cytotrophoblast (JGAS00000000038) [39]and for primed hESCs, PBAT-seq (GSE75868) [40] and RNA-seq (GSE152104) [45] data. Chicken RNA-seq datasets (E-MTAB-7941) [43] for EGK-VIII area pellucida.

### Data processing

RNA-sequencing data was processed with Trim Galore (v0.6.6) and then aligned using HISAT2 (v2.1.0, guided by gene models from Ensembl annotation release 70, options: --dta –sp 1000,1000), hits were filtered to remove mappings with a MAPQ score < 20. PBAT data had the first 9 bp of both read 1 and read 2 removed to reduce biases arising from the 9 N oligo pull-down reaction (Trim Galore options: --clip_r1 9 --clip_r2 0 --paired). PBAT-sequencing libraries were quality and adaptor trimmed with Trim Galore v0.6.6 with -clip parameters. Libraries were deduplicated and methylation calls extracted using PBAT mode in Bismark v0.16.3 [71] with paired-end alignment to the chicken GRCg7b genome assembly.

### Data analysis

Data analysis and figure generation was done using SeqMonk v1.49.2 software. DNA methylation distribution was calculated using 100-CpG windows, which were defined and quantitated with a minimum of 10 informative CpGs for each tissue and time point for each species. Mean DNA methylation was calculated for autosomal 100-CpG windows in mouse (*n* = 206,764), human (*n* = 558,006) and chicken (*n* = 130,688). Gene body DNA methylation was calculated for autosomal genes >5 kb in length with > 10 informative CpGs, removing the first 2 kb of the gene to account for promoter hypomethylation. Gene expression was quantitated using standard RNA quantitation pipeline. Data are presented as log-transformed reads per kilobase of transcript per million mapped reads (Log_2_RPKM). Gene expression deciles were calculated after genes were filtered for length > 5.0 kb, for each trophoblast/EEM dataset in mouse, human and chicken. Pearson’s correlation coefficient was calculated for each species as the correlation between expression level (Log_2_RPKM) and mean gene body % DNA methylation for each gene in each tissue. For genome browser screenshots of RNA-seq, data are quantitated using the Wiggle pipeline in Seqmonk.

DNA methylation of CGIs with a minimum of 10 informative CpGs for mouse, human and chicken, was calculated for each species. The mean of the replicates for each dataset is presented. Binomial filtering was applied to quantitated autosomal CGIs comparing trophoblast/EEM to respective epiblast-derived tissue for each species. CGIs identified as ‘Above’ the model, were filtered for being hypermethylated in trophoblast/EEM compared to epiblast-derived cells. To identify trophoblast/EE-methylated CGIs, these hypermethylated CGIs were then filtered for having less than 10% methylation in epiblast-derived cells, for each species.

Functional annotation analysis was performed using DAVID Bioinformatics [72, 73] (https://david.ncifcrf.gov/). Genes associated with hypermethylated CGIs were annotated as follows: autosomal genes for each species were annotated with the closest CGI with a cutoff of 2 kb. Genes were also then annotated with closest trophoblast/EE-methylated CGI for each species. These gene lists were then analysed using DAVID Ortholog utilising the Orthologous MAtrix (OMA) and Ensembl Compara to retain gene lists featuring only those genes with annotated orthologs in all groups being compared in the analysis. Gene ontology analysis using the GO_BP_DIRECT category, of identified genes was performed using DAVID Bioinformatics, compared to a background population of autosomal genes within 2 kb of a CGI [42], using the *Mus musculus* database.

## Supplementary Information

Supplementary Material 1. Table S1. Omics datasets used in this study. Table S2. Sequencing data generated for this study. Table S3. Significant (Bonferroni p<0.05) Functional Annotation Analysis Terms for Common Mouse and Human Genes Associated with CGIs Hypermethylated in Proliferative Trophoblast. Table S4. Significant Terms for Genes associated with Chicken EE-methylated CGIs also found in Mouse and/or Human.

Supplementary Material 2.

## Data Availability

The bisulphite- and RNA-seq datasets generated for this study are publicly accessible on Gene Expression Omnibus (GSE Accession GSE316283, GSE316284).
