# Supplementary material for: Conserved and divergent DNA methylation features of extraembryonic tissues between human, mouse and chicken
Source: BMC Genomics. 2026 Jul 6;27:788. doi: 10.1186/s12864-026-13123-w (PMC13617923; doi:10.1186/s12864-026-13123-w)
Supplement: Supplementary file 1 [file 12864_2026_13123_MOESM2_ESM.docx]

**Supplementary Figures**

**Supplementary Figure 1. Gene body methylation correlates with transcription in trophoblast cells. a**) Box and whisker plots showing gene body DNA methylation of autosomal genes (>5kb in length), grouped by decile of expression, in mouse E6.5 Epi (left) and ExE (right). **b)** Box and whisker plots showing gene body DNA methylation of autosomal genes (>5kb in length), grouped by decile of expression, in human primed hESCs (left) and 1^st^ CTB (right).

**Supplementary Figure 2. DNA methylation patterns in chicken EEMs at E4 and E14 compared to E8 brain. a)** Box and whisker plots showing DNA methylation within CGIs identified as methylated in E6.5 ExE compared to E6.5 Epi. The plots show that E6.5 Epi hypomethylation of these CGIs is recapitulated in postnatal day 1 (P1) brain, with few outliers. **b)** (Left) Screenshot of an 8Mbp region of mouse chromosome 10, showing DNA methylation as 100-CpG windows in P1 Brain, E6.5 Epi and E6.5 ExE, illustrating the presence of PMDs and gene body methylation in E6.5 ExE only. E6.5 Epi and ExE transcription is also shown across the region. (Right) Screenshot of an 2Mbp region of chicken chromosome 20, showing DNA methylation as 100-CpG windows in E8 Brain, E4 EEM and E14 CAM, illustrating the absence of PMDs and gene body methylation (right). E4 EEM and E14 CAM transcription is also shown across the region. RNA-seq is quantitated using the Wiggle pipeline. **c)** Box and whisker plots showing gene body DNA methylation of autosomal genes, grouped by decile of expression in E4 EEM (top) and E14 CAM (bottom). **d)** Scatter plot comparing autosomal CGI methylation (%) in E4 EEM and E14 CAM. E4 EEM-specific (n=235), E14 CAM-specific (n=369) and commonly-methylated CGIs (n=368) are highlighted. **e)** Box plots showing DNA methylation of common EE-methylated CGIs (n=368) in E8 brain, E4 EEM and E14 CAM. **f)** Pie charts showing that the majority of genes associated with trophoblast-methylated CGIs (mammals) or EE-methylated CGIs (chicken) were lowly transcribed (RPKM<1) in each extraembryonic cell type with a smaller proportion being highly transcribed (RPKM>1).
